# Injection of Xylazine mixed with heroin associated with poor health outcomes and HIV risk behaviors in Puerto Rico

**DOI:** 10.1186/1940-0640-10-S1-A35

**Published:** 2015-02-20

**Authors:** Luz Marilis López, Juliaty Hermanto, Angelee Russ, Deborah Chassler, Lena M Lundgren

**Affiliations:** 1 School of Social Work, Boston University, Boston, MA, 02215, USA

## Background and purpose

Recent studies of injection drug users (IDUs) in Puerto Rico indicate widespread use of xylazine, also known as horse anesthesia (a veterinary analgesic sedative) mixed with heroin and cocaine. Research shows that xylazine contributes to more severe symptoms of withdrawal. This study explored the association between the use of heroin+xylazine and poor health outcomes, including hepatitis C and HIV risk among Puerto Rican drug users.

## Methods

Data from 451 Puerto Rican drug users were gathered by bilingual Spanish and English in-person surveys between 2007 and 2013. Study participants were recruited in areas of San Juan, Rio Piedras, and Ponce where drug users were known to gather. Study eligibility was verified by screening their age (18 or older) and current substance use. Participants received a code based on date of birth and mother’s name to prevent the same respondent from taking the survey multiple times. The study included questions from the Substance Abuse Mental Health Services Administration, Government Performance and Results Act questionnaire and a revised National Institute on Drug Abuse-validated Addiction Risk Behavior Assessment battery. The questionnaire was translated to Spanish, back-translated, and adapted for Puerto Ricans. Bivariate analyses explored the association between heroin+xylazine use and poor health outcomes (self-reported poor health status, being diagnosed with hepatitis C, and HIV risk behaviors). Logistic regression analysis compared the risks associated with using heroin+xylazine and health outcomes, controlling for demographic factors that were significant at the bivariate level.

## Results

Incidence of having injected heroin+xylazine were high (73.4%) and 67.4 percent of the sample had injected xylazine mixed with heroin within 30 days of the interview. Seventy-nine percent of IDUs were male; 54 percent did not have a high school diploma, and 81 percent were homeless or in temporary housing. Heroin+xylazine users were more likely to report lower health status, stating poor or fair health as opposed to good or excellent health (OR = 2.85; CI 1.22–6.66) than those who had never used heroin+xylazine (p < .05). Xylazine mix users were also more likely to report being diagnosed with hepatitis C (OR = 1.90; CI 1.13 – 3.18; p < .05). Substance users who mixed xylazine+heroin were also significantly more likely to have participated in HIV risk behaviors such as injecting more than 5x/day (OR = 9.571; CI 4.72 –19.39); p < .001), sharing works (OR = 2.73; CI 1.34–5.57; p < .05), injecting others (OR = 5.79; CI 2.59–12.98; p = .001), and being injected by others (OR = 5.27; CI 2.36–11.77; p < .001).

## Conclusions and implications

Self-reported poor health outcomes and HIV risk behaviors are significantly associated with heroin+xylazine use. The migratory pattern of Puerto Rican IDUs traveling to and from the USA mainland, possibly carrying xylazine, will likely increase the need for HIV, substance abuse, and hepatitis C prevention efforts in both geographic locations.

